# Case Report: Recurrent acute myocardial infarction in a young woman—the importance of identifying the uncommon underlying causes

**DOI:** 10.3389/fcvm.2025.1527887

**Published:** 2025-05-21

**Authors:** Hong Zhi, Chengyi Xu, Hua Yan, Chengwei Liu, Li Liu

**Affiliations:** ^1^ Department of Cardiology, Wuhan Asia Heart Hospital, Wuhan University of Science and Technology, Wuhan, China; ^2^ Division of Cardiovascular Medicine, Brigham and Women’s Hospital, Harvard Medical School, Boston, MA, United States

**Keywords:** ischemic heart disease, acute myocardial infarction, young woman, Turner syndrome, replacement therapy

## Abstract

Studies have shown that the occurrence and mortality of ischemic heart disease (IHD) in young women aged 35–54 years have increased despite improving trends globally among the general population. Common risk factors such as hypertension, hyperlipidemia, diabetes and smoking play an important role in the occurrence of IHD, but some rare causes that are easily misdiagnosed or undiagnosed should also be paid attention to. Here, we report the case of a young woman (33 years old) who suffered from recurrent acute myocardial infarction (AMI) and was ultimately diagnosed with Turner syndrome (TS) by karyotype testing. TS was identified as the cause of IHD in this patient. We then adjusted treatment strategy to include long-term estrogen-progestin therapy in addition to conventional treatment for IHD (e.g., anti-platelet, lipids-lowering). The patient has been followed up on an outpatient basis and is in good clinical condition. In this report, we highlighted the important of identifying rare causes when treating young women with IHD, and we also discussed the guideline management in such patients.

## Introduction

Although there has been a significant decrease in the mortality of ischemic heart disease (IHD) across the general population over the past few decades, the incidence and mortality of IHD in young women aged 35–54 years continue to increase (1, 2). A observational study from The Atherosclerosis Risk in Communities study found significant trend that young women are having more heart attack (3). While the common risk factors, such as hypertension, high cholesterol, smoking, diabetes and family history of heart disease still play an important role in the development of IHD, some rare causes, such as Turner syndrome, etc., were easily undiagnosed, misdiagnosed or being diagnosed in late childhood or adolescent age, which leads to inappropriate and incomplete management for these patients (4, 5). Turner syndrome is a genetic disorder that affects females only due to complete or partial absence of the second sex chromosome (X chromosome), which occurred in 1 in every 2,000–2,500 live-born girls (5). It can cause a variety of medical and developmental problems. Heart is the most commonly affected organ in patient with TS and the main cause of the early morbidity and mortality, including both congenital cardiovascular defect and acquired cardiovascular conditions (5, 6). Therefore, timely diagnosis is crucial which can initiate early intervention and help TS patients achieve desirable outcomes. Unfortunately, in the real world, most TS patients were diagnosed delayed in China. Here, we report a case of recurrent acute myocardial infarction (AMI) in a young woman (33 years old), the underlying cause of IHD was eventually identified as TS in second emergency hospitalization for AMI, and the patient then received estrogen-progestin therapy in addition to conventional treatment for IHD with good clinical outcome so far. In this report we highlight the importance of careful investigation of rare underlying causes of IHD in young woman and discuss the Guideline management of this rare genetic disease.

## Case presentation

A 33-year-old young woman presented to the hospital with the chief complain of recurrent chest pain after exercising for the past three days. The pain was dull, localized pericardial area, accompanied with palpitation, no radiation and sweeting. The pain can be relieved by rest or nitrates. She denied cough, breathlessness, or syncope. Three months ago, the patient underwent PCI procedure [with the assistance of intra-aortic balloon pump (IABP)] due to “AMI complicated by acute left heart failure and cardiac shock, hyperlipidemia”, when coronary angiography (CA) revealed left anterior descending artery (LAD) subtotal occlusion + the circumflex branch (LCX) subtotal occlusion + right coronary artery (RCA) chronic total occlusion. The patient received PCI, with two stents implanted in LAD and percutaneous transluminal coronary angioplasty (PTCA) with drug-coated balloon for LCX and proximal RCA, however, attempts at distal RCA revascularization failed. Patient was prescribed aspirin (100 mg/day), ticagrelor (90 mg/day), atorvastatin (20 mg/day), ezetimibe (10 mg/day) and followed-up in outpatient clinic. She reported compliance with her medication regimen. She denied hypertension, diabetes, family history of heart disease and any other special medical histories. Upon further inquiry, she admitted that she has no history of menstruation so far. Personal habits included drinking light amounts of alcohol and not smoking.

**Figure 1:**
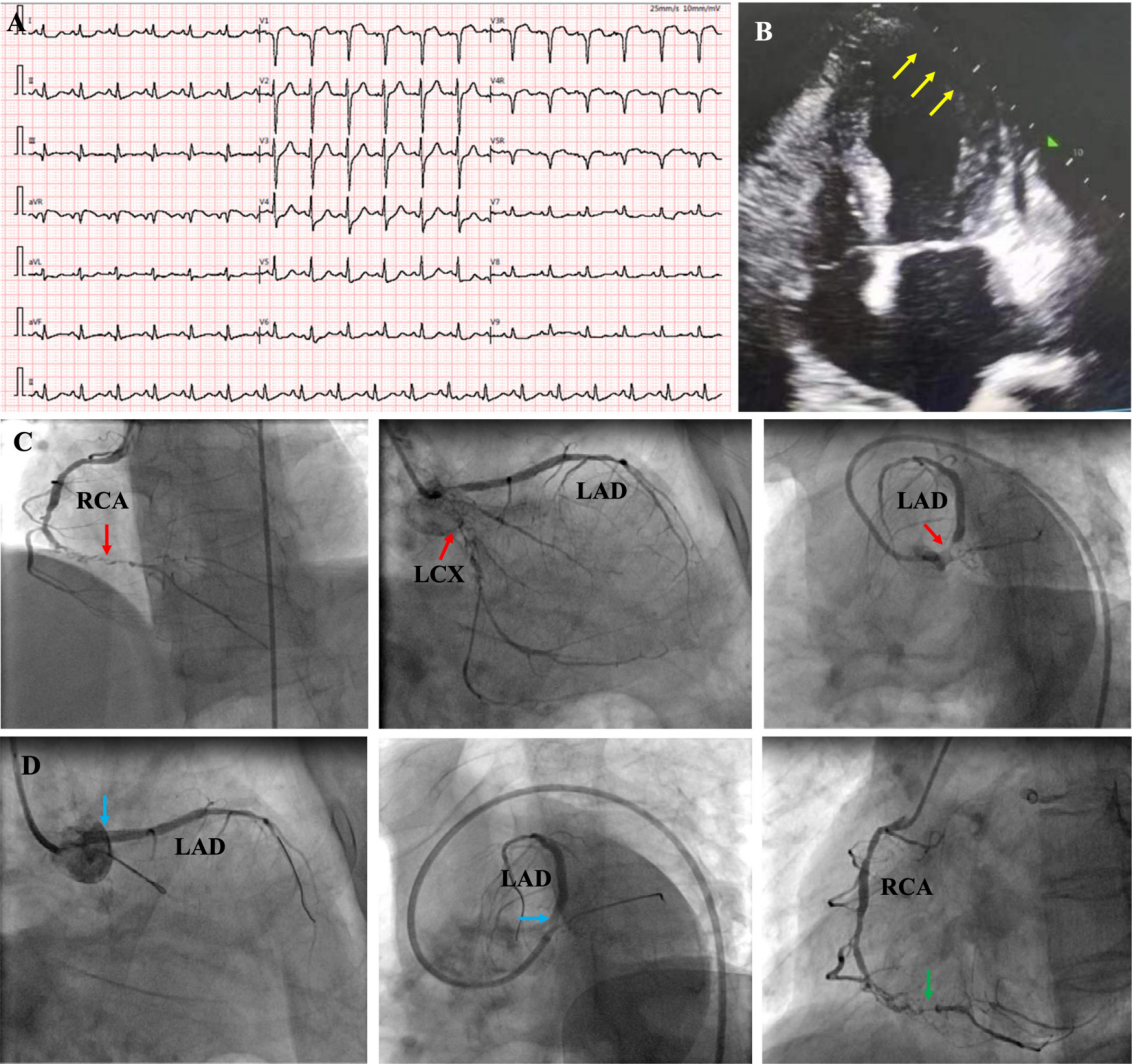
ECG at emergency room showed sinus rhythm with pathological Q-wave on II, III and aVF leads, and T-wave changes on V7–V9 leads **(A)**; echocardiography revealed normal left ventricular (LV) size but reduced left ventricular systolic function (LVEF, 45%), and an akinesis segment in the apical region (size 36 × 13 mm) [**(B)**, yellow arrows]; coronary angiography revealed chronic total occlusion of the right coronary artery (RCA) in the middle segment, and proximal occlusion near the stent in LCX, 95% stenosis in LM and [**(C)**, red arrows]; A stent was implanted in LM-LAD (blue arrow), but the attempt to open LCX and RCA failed [**(D)**, green arrow]. RCA, right coronary artery; LAD, left anterior descending; LM, left main; LCX, left circumflex.

Upon admission, physical examination revealed normal range of blood pressure (113/79 mmHg), heart rate (88 beats/min), respiratory rate (20/min) and oxygen saturation (SO_2_) level (99%, room air). Her height is 148 cm and weight is 55 kg. Cardiac and pulmonary examination revealed normal heart sounds without murmurs and clear lungs. Laboratory tests indicated that complete blood count with differential (CBC w/diff), comprehensive metabolic panel (CMP), hepatic and renal function, and several tumor markers were within normal limit, but elevated cardiac troponin (TNI) 0.287 ng/ml (normal range 0–0.0116 ng/ml) and plasma B-type natriuretic peptide (NT-proBNP) 1186 pg/ml (normal range 0–125 pg/ml). Electrocardiography (ECG) illustrated normal sinus rhythm (HR 88 beats/min) with pathological Q-wave on II, III and aVF leads, and T-wave changes on V7–V9 leads (Figure 1A). Transthoracic echocardiography (TTE) revealed normal left ventricular (LV) size (41 mm) but reduced left ventricular (LV) systolic function [LV ejection fraction (LVEF), 45%], diastolic dysfunction, and an akinesis segment in the apical region (size 36 × 13 mm) (Figure 1B). A diagnosis of “recurrent AMI without ST segment elevation (NSTEMI)” was made and the patient underwent emergency CA, which revealed proximal occlusion near the LCX stent, 95% left main (LM) coronary artery stenosis and distal RCA occlusion (Figure 1C). The patient underwent second PCI, with a stent being implanted in LM-LAD, and attempts to open the LCX and distal RCA occlusion failed (Figure 1D).

**Figure 2:**
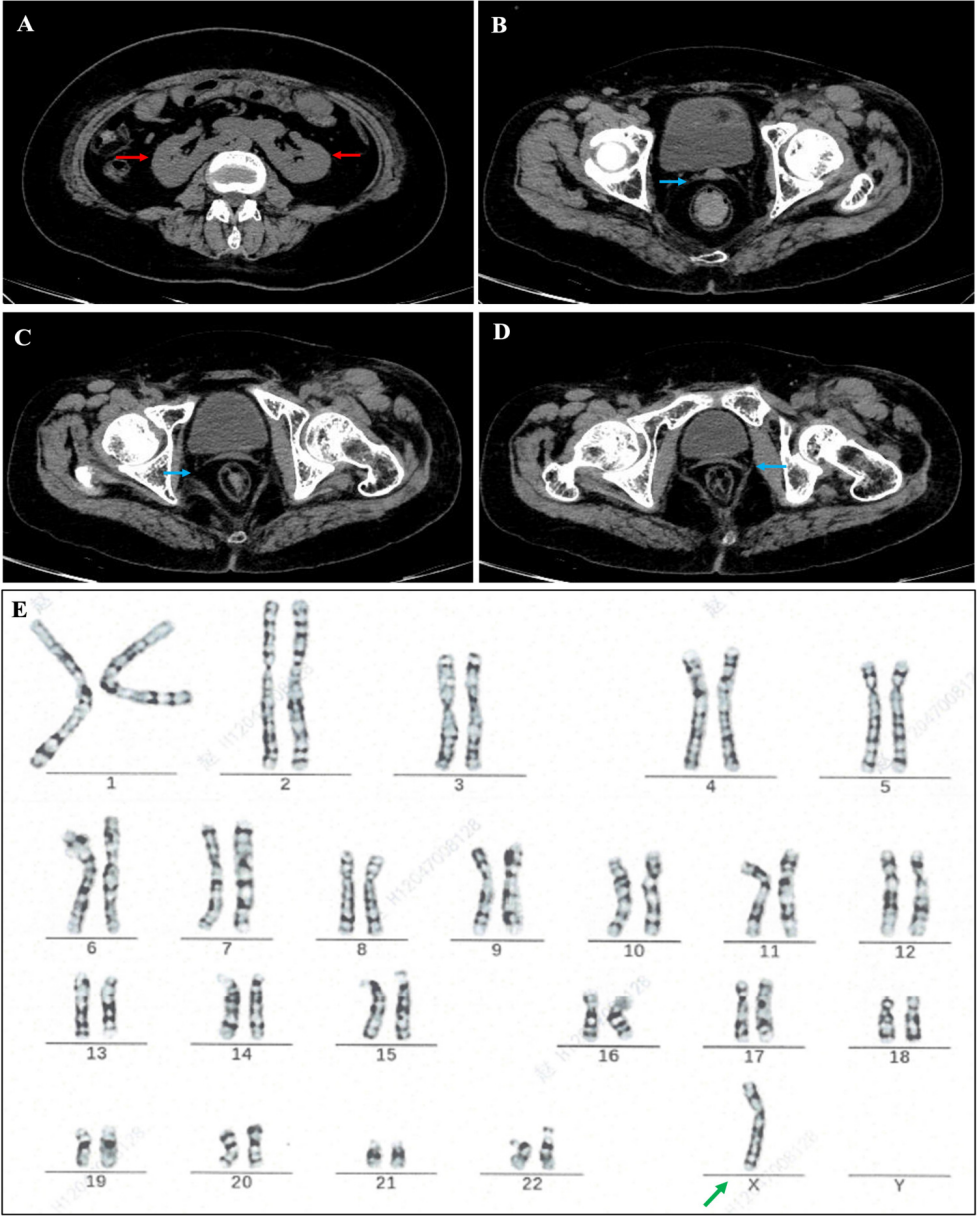
Abdominal computed tomography (CT) showed horseshoe kidney and fused kidney [**(A)**, red arrow], no ovarian structure was seen, and cord-like shadow of uterus was visible [**(B–D)**, blue arrow]; cytogenetic report showing a gene karyotype of 45.X [**(E)**, green arrow].

Since the patient's coronary atherosclerosis progressed rapidly despite ideal treatment of hyperlipidemia (Table 1) and there were no other significant risk factors, we speculated that some unindenting factor(s) may be involved. We then carefully reviewed the patient's physical examination, medical history and laboratory tests, we noticed that the patient was short in stature, had mild intellectual disability and absence of menstruation, the possible diagnosis of Turner syndrome (TS) was suspected. Further investigations, including imaging studies (abdominal CT and pelvic ultrasound) and hormonal tests, were then ordered. Computed tomography showed horseshoe kidneys (Figure 2A) and both CT (Figures 2B–D) and pelvic ultrasonography failed to detect the presence of uterus and ovaries. The thyroid function analysis showed free T3 level 3.03 pg/ml (normal range, 2.3–4.8 pg/ml), free T4 level 1.46 pg/ml (normal range, 0.62–1.24 pg/ml) and thyroid-stimulating hormone (TSH) level 0.01 μIU/ml (normal range, 0.38–5.57 μIU/ml). The level of serum growth hormone was 0.09 ng/ml (reference range 0.016–9.88 ng/ml), the follicle-stimulating hormone was 33.5 mIU/ml (reference range 3.5–12.5 mIU/ml), the testosterone level was <0.02 ng/ml (reference range, 0.084–0.481 ng/ml), the estradiol level <5 pg/ml (reference range 22.3–341 pg/ml), but the level of prolactin and luteinizing hormone was within in normal limit (Table 2). The patient also underwent comprehensive laboratory investigations including erythrocyte sedimentation rate (ESR), immunological workup, thrombophilia screening, anti-neutrophil cytoplasmic antibodies (ANCA), rheumatoid factor panel and anti-cardiolipin antibodies, all test results returned negative. To confirm the diagnosis, we then ordered a genetic test and the result indicated a karyotyping of “45, X” (Figure 2E), which confirmed our diagnosis of TS. We then adjusted the therapeutic strategies to include estrogen and growth hormone therapies in addition to anti-platelet and lipid-lowering therapy. The patient was followed up on an outpatient basis and remained in good clinical condition (>2 years).

**Table 1:** The lipid panel during hospitalization.

Lipid panel	1st hospitalization	2nd hospitalization	Normal range
TC (mmol/L)	7.05	3.93	2.80–5.20
TG (mmol/L)	2.59	1.47	0–1.70
LDL-c (mmol/L)	4.42	2.57	1.00–3.35
HDL-c (mmol/L)	1.20	1.25	0.91–2.60

Patient's lipid panel was monitored during 1st and 2nd hospitalization.

TC, total cholesterol; TG, Triglyceride; LDL-c, low density lipoprotein cholesterol; HDL-c, high density lipoprotein cholesterol.

**Table 2:** Thyroid function and the levels of serum sex hormones.

Hormones	Patient's level	Normal range
Thyroid function		
Free T3 (pg/ml)	3.03	2.30–4.80
Free T4 (pg/ml)	1.46	0.62–1.24
TSH (μIU/ml)	4.42	0.38–5.57
Serum sex hormones		
Growth hormone (ng/ml)	0.09	0.016–9.88
FSH (mIU/ml)	33.5	3.50–12.5
Testosterone (ng/ml)	<0.02	0.084–0.481
Prolactin (ng/ml)	5.5	4.79–23.3
Estradiol (pg/ml)	<5.0	22.3–341
Luteinizing (mIU/ml)	12.80	2.40–12.60

Patient's thyroid function was analyzed and sex hormones were measured during second hospitalization.

T3, triiodothyronine; T4, thyroxine; TSH, thyroid-stimulating hormone; FSH, follicle-stimulating hormone.

## Discussion

Although not completely rare, TS has an estimated prevalence of approximately 1 in 2,000–2,500 live female births (5). The clinical manifestations exhibit considerable heterogeneity, largely dependent on whether the loss of an X chromosome is partial or complete. Patients with mosaic TS may present with milder phenotypes, such as short stature and primary amenorrhea, whereas those with complete loss of an X chromosome (e.g., 45, X) often demonstrate more pronounced physical and psychological impairments. Characteristic clinical features include short stature, facial and oral features, premature ovarian failure and lymphedema of feet and hands (7, 8). Typical facial features include a short, broad neck with webbing, low-set ears, and down-slanted palpebral fissures with epicanthal folds (7). Oral manifestations involve a high-arched palate, hypoplastic mandible, thin enamel and decreased amount of dentin, tooth mobility, and periodontal pockets, prematurely erupted teeth, and various malocclusions (9). Additionally, TS is associated with congenital renal, cardiovascular and thyroid disease (autoimmune hypothyroidism). The most common congenital renal anomaly is horseshoe kidney (8). Girls with TS are at much greater risk for heart disease, including both congenital cardiovascular defect and acquired cardiovascular conditions. Statistics showed ∼50% individuals with TS have congenital heart abnormalities, including bicuspid aortic valve, coarctation of the aorta, and thoracic aortic aneurysm (5, 10). Furthermore, TS patients face an elevated risk of metabolic and cardiovascular comorbidities, such as diabetes mellitus, obesity, dysplipidemia, hypertension (5, 6, 10) or antiphospholipid syndrome (11), all of which contribute to long-term cardiovascular morbidity. The meta-analysis by Siagian et al. (12) elucidates critical risk factors for acute coronary syndrome (ACS) in young women, including diabetes, hypertension, and hypercholesterolemia—all prevalent in TS populations. Our case aligns with these findings, demonstrating how TS-related metabolic dysfunction (dyslipidemia, thyroid disorders) synergizes with estrogen deficiency to accelerate atherosclerosis. Notably, the patient in this case exhibited a complete 45, X karyotype but displayed relatively atypical clinical features, with short stature being the sole prominent manifestation, which is a little different from other case reports (13–15). Additionally, following admission, the patient underwent comprehensive laboratory investigations including erythrocyte sedimentation rate (ESR), immunological workup, thrombophilia screening, anti-neutrophil cytoplasmic antibodies (ANCA), rheumatoid factor panel and anti-cardiolipin antibodies, all test results returned negative, therefore these findings excluded the diagnosis of vasculitides, thrombophilic disorders or rheumatological/autoimmune diseases. The absence of congenital heart anomalies—a hallmark of classical TS—likely contributed to the initial diagnostic oversight during her first hospitalization. Recent advances in diagnostic technologies have significantly improved the accuracy and timeliness of Turner syndrome (TS) detection. Notably, non-invasive prenatal testing (NIPT) utilizing cell-free DNA analysis enables early prenatal diagnosis, while next-generation sequencing (NGS) facilitates the identification of low-level mosaicism and structural X chromosome abnormalities with enhanced precision (16, 17).

Estrogen and growth hormone deficiency due to premature ovarian failure is a significant hallmark in patients with TS. This condition is seen in most patients with TS and may occur at birth or gradually during childhood, adolescence, or early adulthood (18, 19). Patients present as lack of growth, development of secondary sex characteristics (breast, uterine and ovaries) and primary amenorrhea (19, 20). Estrogen deficiency was thought to play a significant role in the non-congenital cardiovascular complications in TS patients. Estrogen has a number of beneficial effects on cardiovascular health. Estrogen modulates vascular function, the inflammatory response, metabolism, insulin sensitivity, cardiac myocyte and stem cell survival through communicating with estrogen receptors (21, 22). Estrogen has potent antioxidant effects and is able to reduce inflammation, induces vasorelaxation and alters gene expression in both the vasculature and the heart (22, 23). Estrogen has a very positive effect on lipoprotein profiles, lowering LDL and raising HDL (24). Conversely, estrogen deficiency disrupts the vascular homeostasis (reduces nitric oxide bioavailability, upregulates adhesion molecules and promotes monocyte infiltration into vascular walls) (21–23) and induces metabolic derangements (elevates LDL cholesterol and causes insulin resistance) (23, 24). These pathological changes contribute to the threefold increase in mortality observed in TS patients compared to the general population, with cardiovascular diseases representing the predominant cause (10). This underscores the critical importance of early diagnosis and intervention in TS management.

While no definitive cure exists for TS, comprehensive management strategies can effectively address its multisystem manifestations. The overarching goals of caring of patient with TS included long-term treatment with estrogen-progestin therapy to prevent adverse consequences of estrogen deficiency including bone loss and increased risk for early coronary heart disease, excess mortality, cognitive decline, and dementia, management of cardiovascular disorders (congenital and acquired), management of fertility issues and potential for pregnancy if desired and possible, surveillance for and management of comorbidities that may include autoimmune thyroid disease, type 2 diabetes mellitus, hearing loss, and abnormal liver enzymes (25, 26). Current therapeutic strategies for TS incorporate a multimodal approach to address growth and cardiovascular complications. The combination therapy of recombinant growth hormone (rGH) with low-dose oxandrolone has emerged as the recommended regimen, demonstrating superior efficacy in achieving final adult height compared to rGH monotherapy (4, 26). Emerging interventions include ovarian tissue cryopreservation in mosaic TS patients prior to ovarian failure, representing a potential fertility preservation strategy, along with prophylactic angiotensin-converting enzyme (ACE) inhibitors to mitigate progressive aortic dilation (4, 26).

Optimal hormone replacement therapy (HRT) follows a carefully staged protocol, with low dose start at age 11–12, gradual dose escalation to mimic physiological puberty, then transiting to full adult replacement doses by approximately 18 years of age. Comprehensive monitoring protocols mandate annual assessment of auxological parameters (height velocity), reproductive development (uterine size via pelvic ultrasound), metabolic profile (fasting lipid panel and glucose homeostasis), bone mineral density (biannual DEXA scanning), cardiovascular status (echocardiography with consideration for cardiac MRI in high-risk cases) (4, 26). In this presented case, the diagnosis was significantly delayed and no congenital heart anormalies were detected, the treatment was tailored to management of acquired cardiovascular complications, including significant dyslipidemia, thyroid dysfunction, atherosclerotic coronary arteries disease, and post-AMI. Longitudinal monitoring revealed no evidence of hepatic dysfunction or glucose intolerance. Following the implementation of estrogen-progestin replacement therapy, the patient has maintained stable clinical status for over 24 months of follow-up, demonstrating the importance of timely hormonal intervention even in atypical presentations.

## Conclusion

AMI occurring in young women should be taken seriously and less common causes should be considered, such as TS. Cardiovascular disease is common in women with TS, resulting in increased mortality in affected individuals. Timely diagnosis and subsequent early intervention can improve quality of life and prognosis. Long-term treatment with estrogen and related hormone therapy can prevent adverse consequences of estrogen deficiency.

## Data Availability

The original contributions presented in the study are included in the article/Supplementary Material, further inquiries can be directed to the corresponding authors.
